# Malaria prevalence in symptomatic and asymptomatic pregnant women in a high malaria-burden state in India

**DOI:** 10.1186/s41182-020-00259-y

**Published:** 2020-08-19

**Authors:** Samir Garg, Mukesh Dewangan, Omprakash Barman

**Affiliations:** State Health Resource Centre, Raipur, Chhattisgarh India

**Keywords:** Malaria, Pregnancy, Community health workers, Screening, India, Febrile, Asymptomatic, Women

## Abstract

**Introduction:**

Malaria in pregnancy (‘MiP’) poses risks to mother, foetus and newborn. Studies from Africa and Asia have reported high prevalence of ‘MiP’ and recommended further research to address ‘MiP’. India has a significant burden of ‘MiP’ but most of the studies are a decade old. Hardly any studies exist in India that report on asymptomatic malaria in pregnant women. The current Indian policies for malaria control are silent on ‘MiP’. A campaign was carried out by community health workers (CHWs) in 2019 to screen pregnant women across rural Chhattisgarh.

**Methods:**

This is a cross-sectional study. Malaria was tested in pregnant women by CHWs using bivalent rapid tests. Multi-stage sampling was used to cover 21,572 pregnant women screened across different geographical areas of rural Chhattisgarh. Cross-tabulation and multivariate regression were used to find out the relationship of ‘MiP’ with different symptoms and geographical areas. GIS maps were used to compare malaria in pregnant women against overall febrile population.

**Results:**

In rural Chhattisgarh, malaria was present in 0.81% of the pregnant women at the time of testing. ‘MiP’ prevalence varied across geographies, reaching 4.48% in the geographical division with highest burden. Febrile pregnant women had three times greater malaria-positivity than overall febrile population and both showed a similar geographical pattern.

**Discussion:**

Prevalence of ‘MiP’ was found to be less than earlier studies in the state. Though overall malaria in India has shown some decline, a policy response is needed for ‘MiP’ in high-burden areas. Fever, diarrhoea and jaundice remain relevant symptoms in ‘MiP’, but around one fourth of malaria-positive pregnant-women were afebrile, suggesting the need for strategies to address it.

**Conclusion:**

The current study based on a large sample provides fresh evidence on ‘MiP’ in India. It used CHWs as skilled providers for large-scale screening for malaria. In high-burden areas, intermittent screening and treatment (IST) of all pregnant women can be a useful strategy in order to address ‘MiP’. Pregnant women can be considered as a pertinent sentinel population for malaria. The global and national policies need to evolve concrete strategies for addressing malaria in pregnancy.

## Introduction

Pregnant women are a group vulnerable to malaria across the globe, especially in areas with high burden of malaria [1–3]. ‘Malaria in pregnancy’ (‘MiP’) poses risks to mother, foetus and newborn, and a large share of malaria in pregnancy can be asymptomatic [1–7]. Studies from many countries of Africa have reported high prevalence of malaria among pregnant women including those without symptoms [8–17]. Studies from Asian countries like Laos and India have also indicated noticeable prevalence of malaria including asymptomatic malaria in pregnant women [18–23]. Asian studies have also emphasised the importance of ‘MiP’ and the need to study it further [7, 24].

The existing studies of prevalence of ‘MiP’ in India have shown moderate prevalence [20–22]. Researchers have emphasised the need for more community-based studies on ‘MiP’ in India, including of efforts through community health workers (CHWs) [20, 23]. There have not been many studies in India that have focused on asymptomatic malaria in pregnancy.

Though concern has been expressed in policy literature over malaria in pregnancy, many countries are yet to decide a clear policy on how to address it in their national programmes. India is a leading contributor to global burden of malaria, but does not have any specific policies on addressing ‘MiP’ [25, 26]. WHO has recommended intermittent preventive therapy (IPT) for high-burden areas [2]. A debate has existed regarding the suitability of intermittent screening and treatment (IST) in comparison with IPT as a strategy for ‘MiP’ [24, 27].

Chhattisgarh is a high burden state in India with Annual Parasite Index (API) of 2.6 [28]. *Plasmodium falciparum* (Pf) is the main form of malaria in Chhattisgarh, followed by *Plasmodium vivax* (Pv). Many areas in the state had API above 10. In 2014, the state decided to start IST for pregnant women during their routine ante-natal check-ups [29]. However, the IST strategy did not get implemented in practice, and no reports were available on IST carried out during routine ante-natal check-ups.

The state had decided other significant measures also in 2014, to detect and treat symptomatic malaria through community health workers (CHWs). The state had 70,000 CHWs known as Mitanins, covering almost all rural habitations in the state [30]. CHWs were trained in detection and treatment of malaria and were equipped with rapid diagnostic tests (RDTs) and anti-malarial drugs. Each CHW looked after a small population of around 300 and had frequent contact with pregnant women [31]. Availability of well-trained CHWs offered another opportunity to implement screening of pregnant women in Chhattisgarh [32]. In 2019, the CHW programme decided to carry out malaria screening of pregnant women .

A special campaign was carried out by CHWs in November, 2019, across rural Chhattisgarh. Under the campaign, CHWs were supposed to carry out the following tasks:

- Test every pregnant woman using WHO-approved bivalent RDTs
- To treat all malaria positive cases in second/third trimesters using ACT for Pf and CQ for Pv
- To refer all malaria-positive cases in first trimester to primary health centres or higher facilities

The above campaign offered an opportunity to study asymptomatic malaria in pregnancy with a sufficiently large sample size.

The objectives of the study were:

- To find out the prevalence of malaria amongst symptomatic and asymptomatic pregnant women
- To compare the geographical distribution of malaria in pregnancy with that of malaria in overall population in rural Chhattisgarh

## Methods and materials

This is a cross-sectional study. Data on each pregnant woman tested was collected from CHWs, who carried out the testing.

### Study setting

**Fig. 1:**
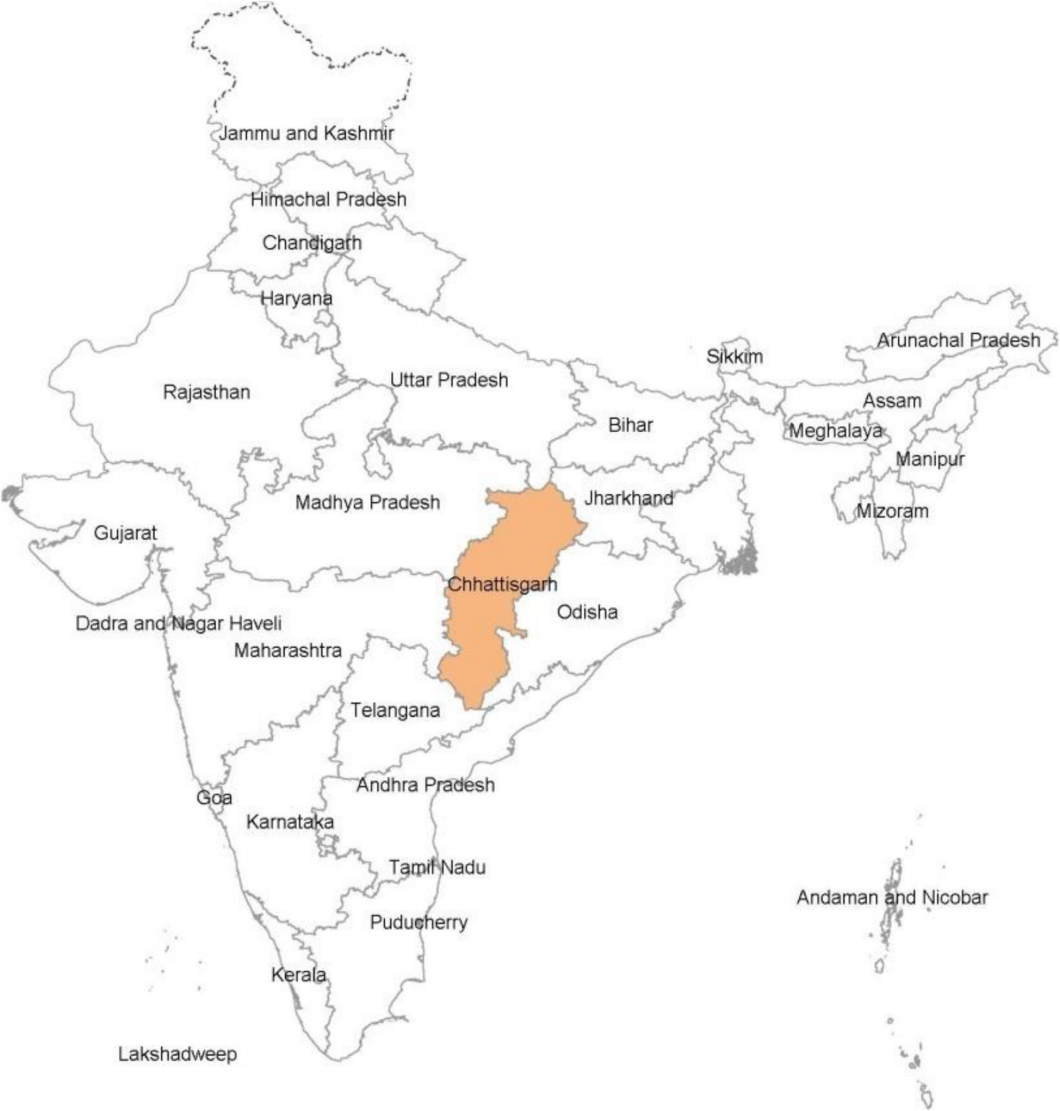
Map of India showing Chhattisgarh and other states

**Fig. 2:**
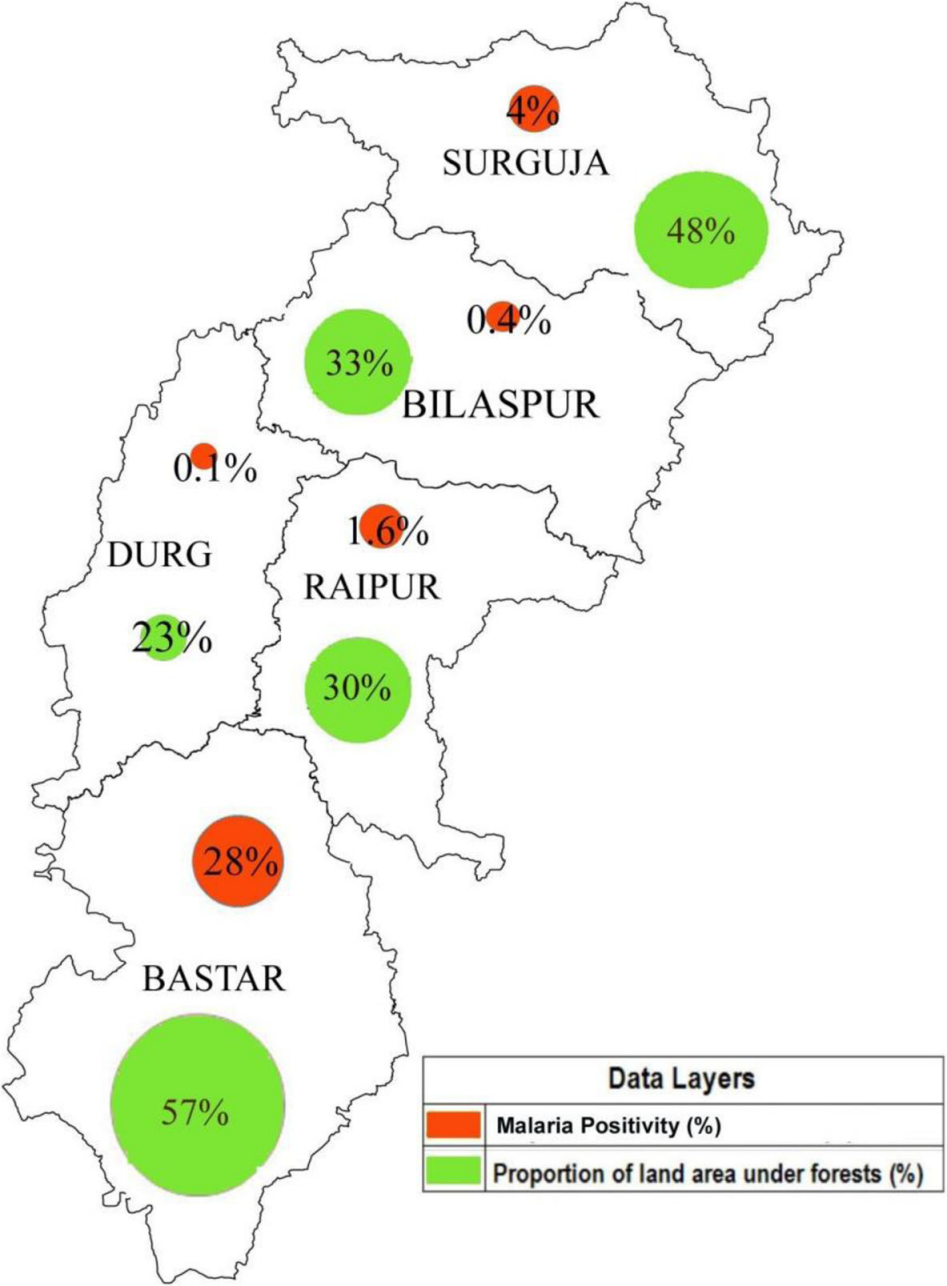
Map of Chhattisgarh showing different administrative divisions and key indicators. Sources of data: forest cover [33] and malaria positivity [35]

Chhattisgarh is one of the younger states in India, formed in year 2000. A map of India showing its states including Chhattisgarh is given in Fig. 1. Forty-one percent of the state’s geographical area is covered with forests [33]. The state has five administrative divisions. The Sarguja division in the north and the Bastar division in the Southern part of the state are highly forested. These areas have a history of high malaria incidence [34]. The central region of the state consists mainly of the agricultural plains and has divisions of Bilaspur, Raipur and Durg. A map of Chhattisgarh state with its administrative divisions and their forest-cover and malaria positivity is given in Fig. 2.

### Screening method

Malaria was tested in pregnant women by CHWs using bivalent RDTs, approved by Indian Government and WHO [26, 36].

### Ethics and consent

Ethics approval was obtained from the Institutional Ethics Committee of State Health Resource Centre, Chhattisgarh. Written consent was obtained from pregnant women for testing and data collection. The dataset was anonymised. Consent was obtained from the CHW programme for the data used and published in this study.

### Sampling

A sample size requirement of around 20,000 pregnant women was estimated. The study applied stratified multi-stage random sampling. Each of the five administrative divisions in the state has 24 to 32 administrative blocks. Blocks are smaller administrative units with average population of around 100,000. For the study, four blocks were randomly selected from each of the five divisions. In each of the 20 blocks thus selected, data was collected on all pregnant women tested by CHWs during November–December, 2019. The study was able to collect data on 21,572 pregnant women who had got tested by CHWs in the 20 sample blocks.

### Study variables

Fever is a well-known symptom of malaria [2]. Diarrhoea has also been reported as a common symptom in malaria [37, 38]. Some studies have reported jaundice to be common among malaria cases [39]. The above three were taken as the relevant symptoms. The screening drive took place during winter months of 2019. Fever was taken as temperature above 37 °Celsius, as recorded by CHWs at the time of testing. Jaundice was assessed clinically by CHWs at the time of testing.

Prevalence of malaria was measured as proportion of the tested individuals found as malaria positive. This has also been referred as malaria positivity in this article. Trimester of pregnancy was a variable. Administrative division was taken as a variable for geography.

For comparing ‘MiP’ with the malaria in overall febrile population, secondary data on block-wise testing done by CHWs during the same period as the study (November–December 2019) was obtained from the CHW programme [35].

### Data analysis

Geographical information system (GIS) maps were used to show malaria prevalence across geographical areas and to compare that between pregnant women and overall febrile population. This was done using software called Map Window (version 5), an open source desktop GIS application [40]. The blocks in different quintiles of malaria prevalence were shown in different colours. This method was used, as spatial patterns can be better represented through maps.

Descriptive statistics was used to report key indicators of malaria and different symptoms. Cross-tabulations were carried out. For key indicators, 95% confidence intervals were reported. Multivariate analysis was carried out to find out the association between malaria-positive cases and different variables. Logistic regression was carried out with malaria positivity as the outcome variable. The model included variables related to geography, the trimester of pregnancy and symptoms at time of testing because the objective was to find out their association with malaria positivity. For geography, administrative division was taken as a variable. For symptoms, variables of fever, diarrhoea and jaundice were included. Data was analysed using STATA version 15.

## Results

The study was able to cover an average of 7.7 pregnant women per 1000 rural population. This constitutes around half of the expected number of pregnant women in the population.

### Malaria prevalence in pregnant women

The state-level prevalence of ‘MiP’, i.e. the proportion of pregnant women who tested positive for malaria at the state level, was 0.81%. The prevalence of ‘MiP’ was unequal across divisions with the Bastar division having the highest rate (4.48%) followed by the Sarguja division (1.04%). Prevalence in the other three divisions was much lower at 0.19%, 0.27% and 0.03% for Bilaspur, Raipur and Durg, respectively.

### Malaria prevalence in symptomatic and asymptomatic pregnant women

**Fig. 3:**
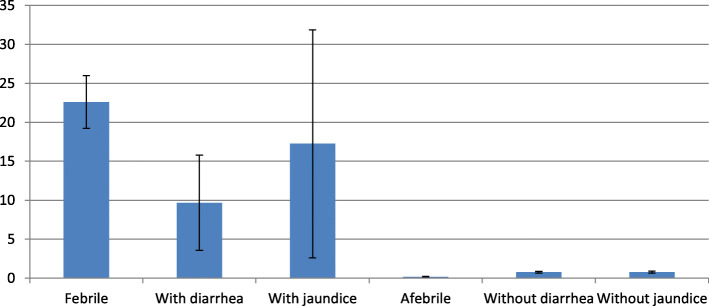
Malaria prevalence in pregnant women at state level—with and without symptoms (% malaria positive with 95% CI)

The prevalence of malaria was higher among the febrile as compared to the afebrile pregnant women. A similar pattern was observed in the comparison of malaria prevalence among pregnant women having diarrhoea or jaundice versus those without these symptoms (Fig. 3).

Afebrile malaria cases constituted 23.3% (CI 17–29.6%) of all malaria-positive cases. Afebrile malaria cases were found mainly in the high-burden divisions of Bastar and Sarguja.

### Comparison of geographical pattern of malaria in pregnant women against malaria in overall population

**Fig. 4:**
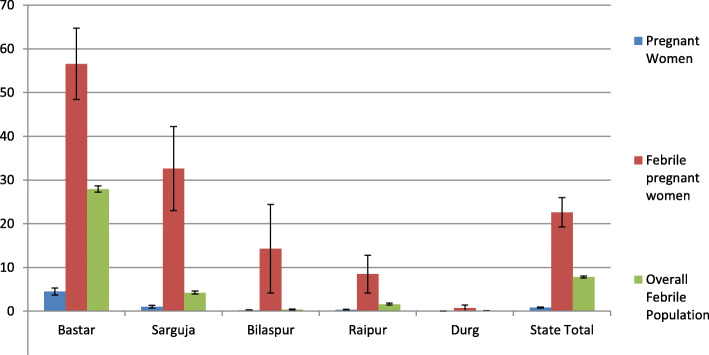
Division-wise comparison of malaria positivity in pregnant women versus overall febrile population in rural Chhattisgarh November–December 2019 (% malaria positive with 95% CI)

Figure 4 compares the prevalence of malaria in pregnant women and the overall population. It shows that the geographical pattern of malaria across divisions was similar for pregnant women and the overall population. The geographical areas having high prevalence of malaria in pregnant women also had high prevalence of malaria in overall population. It showed that malaria prevalence amongst febrile pregnant women was around three times the prevalence in overall febrile population.

The block-wise comparison of malaria positivity in pregnant women against the malaria positivity in overall population during the same period showed a strong correlation between the two (Pearson correlation = 0.90, *p* < 0.001) [35].

**Fig. 5:**
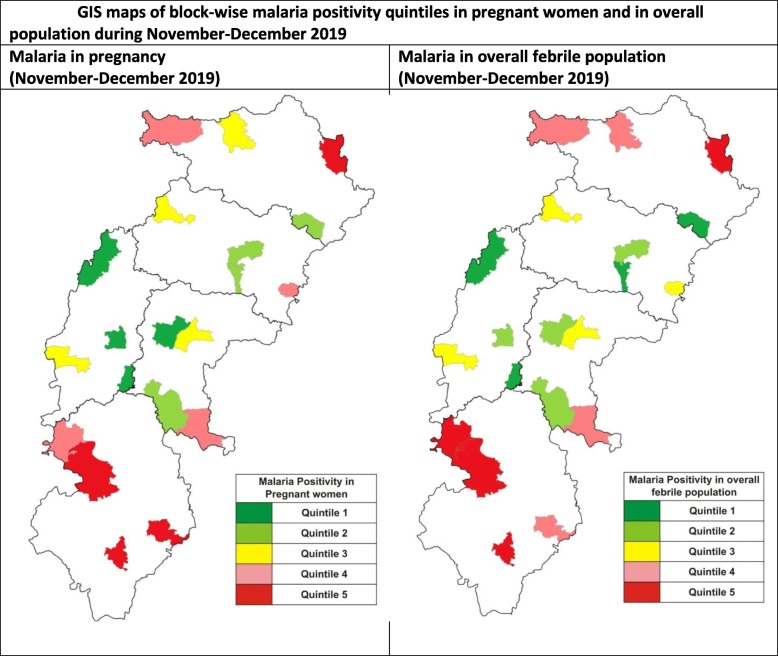
GIS maps of block-wise malaria positivity quintiles in pregnant women and in overall population during November–December 2019

Figure 5 provides GIS maps of malaria positivity in pregnant women against the malaria positivity in overall population in the study blocks during November–December 2019. The blocks falling under different quintiles have been shown in different shades. Quintile 1 has the lowest malaria-positivity rate, and quintile 5 has the highest. Figure 5 indicates that the two share a similar geographical pattern. Geographical areas found with high malaria prevalence for both the groups were those with greater forest cover and a history of high malaria prevalence.

### The association between geography, symptoms, trimester and malaria positivity

The model of logistic regression to find out predictors of malaria positivity included geographical variable of administrative division, apart from the trimester and symptoms at time of testing (Table 1).

**Table 1:** The association between geography, symptoms, trimester and malaria positivity

Malaria positive		Odds ratio	(95% CI)	***P*** > (Z)
Trimester of Pregnancy	First trimester	1		
	Second trimester	1.01	.62 to 1.64	0.96
	Third trimester	.95	.57 to 1.57	0.83
Fever	No	1		
	Yes	141.52	95.39 to 209.95	< 0.01***
Jaundice	No			
	Yes	1.57	.43 to 5.66	0.49
Diarrhoea	No	1		
	Yes	3.52	1.15 to 10.80	0.03**
Division	Bastar	1		
	Sarguja	.27	.17 to .41	< 0.01***
	Bilaspur	.07	.03 to .15	< 0.01***
	Raipur	.046	.02 to .08	< 0.01***
	Durg	.01	.002 to .027	< 0.01***
	Cons	.01	.007 to .017	< 0.01

*p < 0.1, **p < 0.05, ***p < 0.01

The above model shows that fever and diarrhoea among the symptoms and geography as represented by administrative divisions were significantly associated with malaria prevalence.

## Discussion

Compared to other nations, considerable literature is available on ‘MiP’ in Africa [12]. The prevalence rates of ‘MiP’ reported from many African countries have been high—18 to 40% in Burkina-Faso, 32% in Zambia, 29% in Congo, 27% in Uganda, 13% in Tanzania, 12% in Liberia and 4% in Ethiopia [8–17]. Compared to Africa, prevalence was found to be lower in Asian countries. It was found to be around 6% in Laos and 0.4% in Afghanistan [18, 19]. Studies from India had reported ‘MiP’ prevalence of 1.3% in Chhattisgarh, 1.8% in Jharkhand and 6.4% in Madhya Pradesh state [20–22].

The current study found 0.81% of pregnant women to be malaria-positive. A study around a decade earlier had reported a corresponding figure of 1.3% in Chhattisgarh [20]. This suggests that malaria-prevalence in pregnancy has declined in the state. Data from national programme on malaria had also shown a decline in malaria incidence in recent years [28].

The malaria positivity among the afebrile pregnant women was 0.19% in the current study whereas earlier studies had reported greater prevalence—0.5% in Chhattisgarh and 1.1% in Jharkhand [20, 21]. The current study found that afebrile malaria still constituted around one fourth of malaria positive cases in pregnancy. Additional measures are needed to address it.

The current study examined ‘MiP’ detection through RDTs and found it suggested that community-based screening using RDTs was a feasible strategy. Other studies had shown the advantages of RDTs including their cost-effectiveness [12, 16, 41]. A qualitative study had shown that formal providers like nurses had greater trust in microscopy and some doubted whether RDTs gave correct results [42]. The current study did not indicate such issues in context of CHWs carrying out large-scale screening using RDTs. The experience in Chhattisgarh suggested better feasibility of IST through CHWs as compared to formal providers. A study in Chhattisgarh and Jharkhand a decade ago had found that formal health workers did not perceive ‘MiP’ as an important problem and were poorly equipped to address it [43]. CHWs, when trained and equipped adequately, are known to enjoy trust of local communities and offer the important advantage of being close to community [44].

A recurring theme in literature on ‘MiP’, from both Africa and Asia, has been of the perceived apprehensions associated with taking anti-malarial drugs during pregnancy [42, 45–48]. Such fears had posed challenges for IPT but to a lesser extent for IST. One study from Ghana found IST to be more acceptable than IPT [46]. The fear of needle prick in IST was not found to be a big barrier. IST was found to be acceptable to pregnant women and providers in Indonesia [42, 47]. The acceptability of IST was found to be high where the providers enjoyed trust of community, especially women [27]. Other important factors identified for successful IST included good availability of RDTs with close to community providers and training of providers with well-designed messages on IST [44, 48].

Some studies have recommended vector-based measures, especially insecticide-treated bed-nets, as a strategy for addressing ‘MiP’ [8, 20, 21]. A study in Uganda recommended use of either IPT or IST in a context with high prevalence of ‘MiP’ [13]. A study from Burkina Faso recommended active screening in order to address the high burden of ‘MiP’ [9]. Further studies on IST have been recommended in Africa [12]. Studies from other regions of the globe have also recommended IST as well as further research on ways to address ‘MiP’ [24].

IST has been suggested for addressing ‘MiP’ in many contexts but is not firmly present in national policies or WHO’s recommendations [1, 2]. A review of ‘MiP’ has recommended IST, especially for areas where effectiveness of IPT has been reduced by drug-resistance [12]. A study from Tanzania recommended a shift from IPT to IST, considering the availability of RDTs as a cost-effective tool [41]. A simulation study showed that IST could be a cost-effective alternative to IPT especially where drug-resistance is high [49]. It has been recommended that addressing ‘MiP’ can help in reducing the problem of post-partum malaria also [50].

Current Indian policies on malaria have not included any specific strategies like IST or IPT for ‘MiP’ [25, 26]. Earlier studies of ‘MiP’ in India have concluded that its prevalence was low, around 2% [20, 21]. They have suggested insecticide-treated bed-nets and other vector based strategies and IST [20, 21]. With easy availability of RDTs, IST can be recommended for ‘MiP’ especially where well-trained close to community providers are available. The current study found that the febrile pregnant women had three times the malaria prevalence as compared to the overall febrile population in rural Chhattisgarh. In areas with high API, strategy of IST should be considered for addressing ‘MiP’.

It has been debated whether pregnant women should be considered as a sentinel population for malaria surveillance [17, 51]. The current study found that the geographical pattern of malaria in pregnancy was similar to malaria in overall population, suggesting that screening of pregnant women can help in identifying areas having high burden of malaria, including asymptomatic malaria.

### Limitations

The CHWs were able to cover around half of the pregnant women in screening. The current study did not collect data on gravida which others have found to be a relevant factor in ‘MiP’ [6]. The study did not examine relationship of ‘MiP’ with socio-economic characteristics of pregnant women and focused on geography and symptoms instead.

## Conclusions

The study examined the prevalence of malaria and some key symptoms in a screening drive targeted at pregnant women. It used CHWs as skilled providers for large-scale screening of malaria. Intermittent screening and treatment (IST) of all pregnant women in high-burden areas should be part of the strategy to address malaria in pregnancy. Pregnant women can be considered as a pertinent sentinel population for malaria. The global and national policies need to evolve concrete strategies for addressing malaria in pregnancy.

## Data Availability

The datasets used and/or analysed during the current study are available from the corresponding author and State Health Resource Centre, Chhattisgarh on reasonable request.

## Abbreviations

ACT: Artemisinin combination therapy
API: Annual Parasite Index
CHW: Community health workers
IPT: Intermittent preventive therapy
IST: Intermittent screening and treatment
LMIC: Low- and middle-income countries
‘MiP’: Malaria in pregnancy
Pf: *Plasmodium falciparum*
Pv: *Plasmodium vivax*
RDT: Rapid diagnostic test
